# Label-Free Infrared Spectral Histology of Skin Tissue Part II: Impact of a Lumican-Derived Peptide on Melanoma Growth

**DOI:** 10.3389/fcell.2020.00377

**Published:** 2020-05-29

**Authors:** Stéphane Brézillon, Valérie Untereiner, Hossam Taha Mohamed, Estelle Ahallal, Isabelle Proult, Pierre Nizet, Camille Boulagnon-Rombi, Ganesh. D. Sockalingum

**Affiliations:** ^1^Université de Reims Champagne-Ardenne, Laboratoire de Biochimie Médicale et Biologie Moléculaire, Reims, France; ^2^CNRS UMR 7369, Matrice Extracellulaire et Dynamique Cellulaire - MEDyC, Reims, France; ^3^Université de Reims Champagne-Ardenne, PICT, Reims, France; ^4^Zoology Department, Faculty of Science, Cairo University, Giza, Egypt; ^5^Faculty of Biotechnology, October University for Modern Sciences and Arts, Giza, Egypt; ^6^CHU de Reims, Laboratoire Central d'Anatomie et de Cytologie Pathologique, Reims, France; ^7^Université de Reims Champagne-Ardenne, BioSpecT-EA7506, Reims, France

**Keywords:** melanoma, lumican-derived peptides, B16F1, infrared histology, immunohistochemistry

## Abstract

Melanoma is the most aggressive type of cutaneous malignancies. In addition to its role as a regulator of extracellular matrix (ECM) integrity, lumican, a small leucine-rich proteoglycan, also exhibits anti-tumor properties in melanoma. This work focuses on the use of infrared spectral imaging (IRSI) and histopathology (IRSH) to study the effect of lumican-derived peptide (L9Mc) on B16F1 melanoma primary tumor growth. Female C57BL/6 mice were injected with B16F1 cells treated with L9Mc (*n* = 10) or its scrambled peptide (*n* = 8), and without peptide (control, *n* = 9). The melanoma primary tumors were subjected to histological and IR imaging analysis. In addition, immunohistochemical staining was performed using anti-Ki-67 and anti-cleaved caspase-3 antibodies. The IR images were analyzed by common K-means clustering to obtain high-contrast IRSH that allowed identifying different ECM tissue regions from the epidermis to the tumor area, which correlated well with H&E staining. Furthermore, IRSH showed good correlation with immunostaining data obtained with anti-Ki-67 and anti-cleaved caspase-3 antibodies, whereby the L9Mc peptide inhibited cell proliferation and increased strongly apoptosis of B16F1 cells in this mouse model of melanoma primary tumors.

## Introduction

Melanoma is the most aggressive and deadliest form of skin cancers representing 80% of deaths in cutaneous malignancies (Miller and Mihm, 2006; Hodi et al., 2010). The early stages of melanoma can be cured *via* surgery. In contrast, treatment of metastatic melanoma is a health issue due to resistance to most available therapies and low survival rates (Soengas and Lowe, 2003; Gray-Schopfer et al., 2007; Greene and Sobin, 2008). The biological changes occurring in the primary tumor that lead to metastatic tumors including loss of adherent junctions, extracellular matrix (ECM) degradation, increased carcinoma cells motility and resistance to apoptosis, are now better understood. In addition, the important role of stromal and infiltrating immune cells in tumor progression and patient's outcome has been reported in several studies (Pages et al., 2010; Hanahan and Weinberg, 2011; Galon et al., 2012). Thus, anticancer strategies developed over the last years, focused on understanding the cross-talk between malignant cells (Valkenburg et al., 2018; Liu et al., 2019) and the tumor microenvironment including both stromal cells and ECM (Belotti et al., 2011; Venning et al., 2015) such as targeting matricellular proteins, that regulate the communication between ECM and cancer cells (Wong and Rustgi, 2013).

Lumican belongs to the small leucine-rich proteoglycans (SLRP) family (McEwan et al., 2006) and was shown to control the assembly of collagen fibers in the ECM (Chakravarti et al., 1998; Iozzo and Schaefer, 2015). Proteoglycans play a major role in the control of tumor progression. Lumican is expressed in various tumor tissues but both positive and negative correlations with tumor aggressiveness have been reported (Brézillon et al., 2013; Nikitovic et al., 2014). Brézillon et al., revealed that the downregulation of lumican expression in melanoma is associated with increased invasion (Brézillon et al., 2007). They have also shown that lumican inhibits melanoma cell migration (Brézillon et al., 2009; Stasiak et al., 2016), while promoting their adhesion (D'Onofrio et al., 2008). Moreover, previous studies showed the ability of lumican (and its derived peptides), in contrast to decorin, to inhibit MMP-14 activity in melanoma cells, where lumican directly interacts with MMP-14 and inhibits its activity (Pietraszek et al., 2013, 2014). Thus, lumican is able to inhibit the remodeling of the skin ECM by inhibiting MMPs activity, and consequently melanoma progression. Furthermore, lumican is highly expressed within the stroma surrounded several solid tumors such as prostate cancer (Coulson-Thomas et al., 2013) and lung adenocarcinoma (Cappellesso et al., 2015). Another study reported that extracellular lumican enhances the cytotoxicity of chemotherapy in pancreatic ductal adenocarcinoma cells by autophagy inhibition (Li et al., 2016). Recently, optical imaging techniques such as Second Harmonic Generation (SHG) and FTIR were used to validate that lumican disorganizes ECM and more specifically collagen fibers orientation (Jeanne et al., 2017).

IR spectroscopy is a promising rapid, non-destructive, reagent and label-free technique that is used for structural and compositional analysis due to its ability to give a complete “molecular fingerprint” of the studied sample (Baker et al., 2014). It is highly sensitive to the structure, composition, and environment of the molecules constituting the studied specimen. It has been successfully used to characterize, differentiate, and classify types and subtypes of glycosaminoglycans (GAGs) despite their close molecular structures (Mainreck et al., 2011; Mohamed et al., 2017) and to perform cell phenotyping (Brézillon et al., 2014, 2017; Mohamed et al., 2017, 2018).

At the tissue level, FTIR imaging combined with multivariate statistical analysis has shown its capability to discriminate between inflammatory and non-inflammatory breast cancer tissues (Mohamed et al., 2018). Wald et al., demonstrated that FTIR imaging histopathology was able to discriminate between non-metastatic and metastatic lymph nodes from melanoma patients and to distinguish between infiltrating lymphocytic cell subpopulations, thus predicting if they originated from normal or metastatic lymph nodes (Wald et al., 2016a). However, they did not find any significant differences between primary and metastatic melanoma cells or any significant correlation between the infrared spectra of melanoma cells and the percentage of proliferative cells (Wald and Goormaghtigh, 2015).

In the present report, we conducted an investigation combining IR spectral histopathology (IRSH), conventional histology and immunohistochemistry to study the effect of lumican-derived peptide (L9Mc) on tumor progression in melanoma primary tumors.

## Materials and Methods

### Cell Culture

Murine melanoma cell line B16F1 (ATCC®CRL-6323™) was cultured in DMEM medium with 10% fetal bovine serum, 1% of penicillin/streptomycin antibiotic mixture and 50 mg/mL geneticin at 37°C and 5% CO_2_.

### *In vivo* Studies

A total of 27 female C57BL/6 mice were purchased from Harlan-France (Gannat, France) for enrolment in this study. Mice were individually caged in a room with fixed level of humidity and temperature. For mice nutrition, we used standard food and water. All mice were adapted for 7 days before starting the experiments. B16F1 cells at 80% confluency were detached using trypsin/EDTA solution, then the cell suspension was centrifuged at 300 g for 3 min. The collected cell pellet was resuspended in basal medium at the concentration of 2.50 × 10^6^ cells/mL in absence or presence of 100 μM lumican-derived peptide L9Mc, or its scrambled peptide (L9Mc SCR). At day 0, 25 × 10^4^ B16F1 cells (mixed or not with the peptides, 2 mg/mL) were injected in the right flank of 27 mice (*n* = 9 for control, *n* = 8 for L9Mc SCR and *n* = 10 for L9Mc). In addition, 200 μg of peptides at 2 mg/mL were injected at days 6, 9, and 13 to the corresponding groups. At day 19, mice were sacrificed and the primary tumors were collected and formalin-fixed paraffin-embedded (FFPE) for conventional histology, immunohistochemistry, and IRSH. All experiments were performed according to the instructions of the Center National de la Recherche Scientifique. This study was performed in compliance with the French Animal Welfare Act and following the French Board for Animal Experiments. Experiments were conducted under approval of the French “Ministère de l'Enseignement Supérieur et de la Recherche” (Ethics Committee C2EA-56) in accordance with the directive “2010/63/UE.”

### Histopathological Examination of Skin Tissue Samples

Three 5 μm thick serial sections were obtained from the FFPE tumor tissues. The first and third sections were chemically dewaxed and stained with standard Hematoxylin and Eosin solution (H&E) that respectively highlights the nucleus in purple and the cytoplasm in pink. These sections underwent histopathological examination by a confirmed pathologist from the Pathology Department of the Reims University Hospital to annotate the different tissue structures.

### Immunohistochemistry

Immunohistochemical (IHC) staining was performed after chemical dewaxing of two 3 μm thick sections using antibodies against Ki-67 (SP6) (Abcam, Cambridge, UK) and cleaved caspase-3 (Cell Signaling, Danvers, MA, USA). Ki-67 and cleaved caspase-3 stainings are indicators for proliferation and apoptosis indexes, respectively. IHC staining was carried out by adding 100 μL of DAB+chromogen diluted at 1:50 in substrate buffer [EnVision+ Dual Link System-HRP (DAB+)] for 10 min. Finally, tissue specimens were washed in phosphate buffer saline (PBS), the nuclei counterstained with hematoxylin and mounted using Eukitt® for microscopic examination. Positively stained melanoma cells, in which Ki-67 and cleaved caspase-3 highlight in brown the nucleus and the cytoplasm respectively, were quantified using ImageJ software (National Institutes of Health, Bethesda, MD, USA).

### Infrared Spectral Imaging of Formalin Fixed Paraffin Embedded Tissues

The second 5 μm thick tissue section was placed on a 1 mm thick calcium fluoride (CaF_2_) window (Crystran, Dorset, UK) for IRSH analysis without any particular preparation such as chemical dewaxing or staining. FTIR images were acquired in transmission mode using the Spotlight™ 400 imaging system (PerkinElmer, Villebon-sur-Yvette, France) at a pixel size of 6.25 μm, a spectral resolution of 4 cm^−1^ and 16 scans in the spectral range of 800–4,000 cm^−1^. Prior to this, a visible image of the tissue section is acquired using the IR microscope and the region of interest selected with the help of the H&E stained tissue. Further, a background spectrum was recorded in a blank area of the window that was automatically subtracted from each pixel spectrum of the image.

### Spectral Image Preprocessing

**Figure 1:**
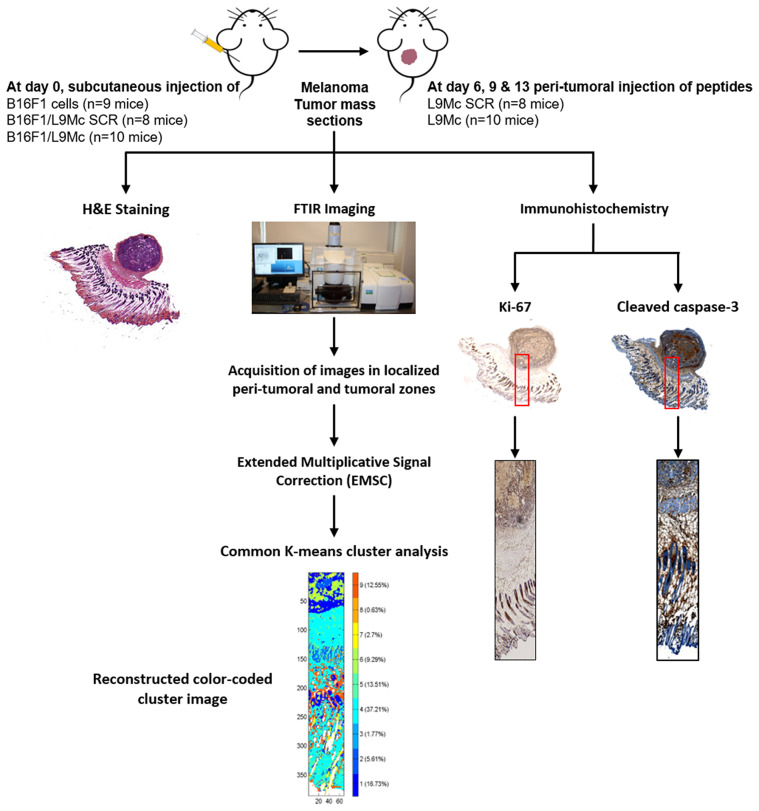
Workflow showing the histology, FTIR imaging analysis, and immunohistochemistry of FFPE melanoma sections, and analysis of FTIR images with common K-means clustering.

The recorded FTIR hyperspectral images of FFPE melanoma tissues exhibit both tissue biochemical information and paraffin bands (1,378 and 1,467 cm^−1^) in the 900–1,800 cm^−1^ spectral region. To avoid chemical dewaxing, images were digitally corrected for paraffin spectral contribution. This is achieved *via* an automated data processing method based on Extended Multiplicative Signal Correction (EMSC) as reported before (Ly et al., 2008). To obtain IRSH images, an unsupervised common K-means clustering method was applied to the tissue spectra (Nguyen et al., 2016). In this method, each spectrum belongs to a unique cluster and spectral images can be reconstructed based on pixel spectral similarity for a rapid and simple visual analysis of clustering results. Both EMSC and K-means clustering algorithms were implemented in Matlab Statistics Toolbox software. In addition to the cluster images, a dendrogram obtained by Hierarchical Cluster Analysis (HCA) and based on spectral distance calculation between different cluster centroids, was also obtained. Each centroid spectrum can be assigned to a different tissue component. All processed IRSH images were compared with adjacent H&E and IHC stained sections. The workflow for histopathological, immunohistochemical, and IRSH analyses is illustrated in Figure 1.

### Statistical Analysis

All data are presented as the mean ± standard deviation (SD). One-way ANOVA was used in the three groups comparison analysis of melanoma primary tumors data. In addition, Student's *t*-test was used in a pairwise comparison analysis of melanoma primary tumors data. A *P* < 0.05 was considered significant with ^*^P < 0.05 and ^**^P < 0.01.

## Results

### Infrared Spectral Histology Correlates Well With Conventional Histology of Melanoma

**Figure 2:**
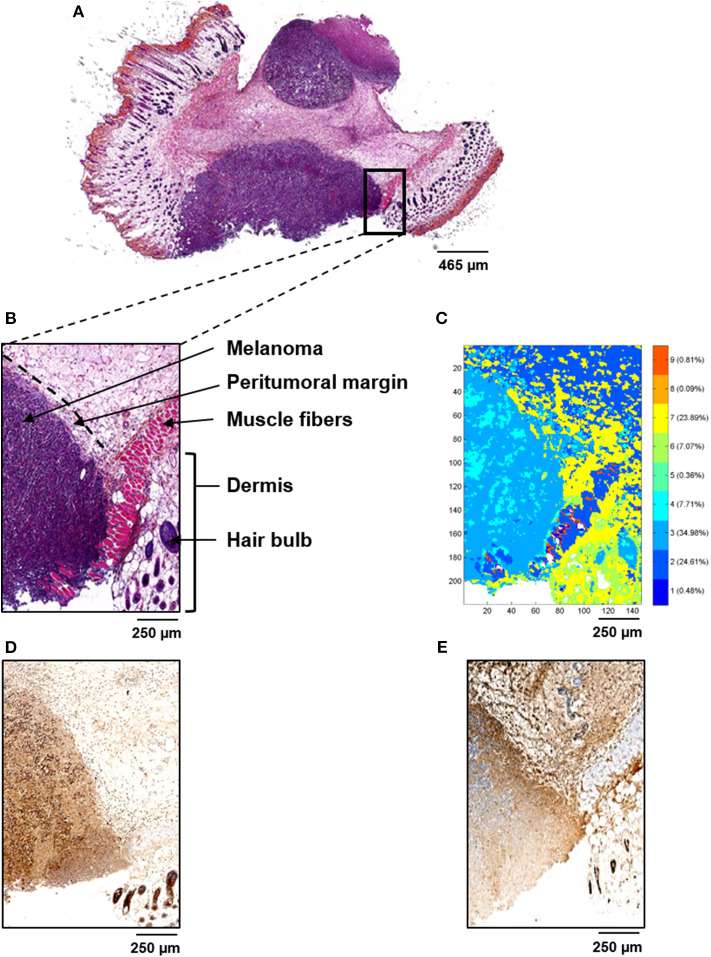
Conventional and spectral histologies of control melanoma primary tumor tissues. **(A)** H&E staining of the whole tissue sample. **(B)** Selected ROI (represented by a black rectangle in **(A)** used for FTIR imaging. **(C)** Common K-means FTIR reconstructed image using 9 classes revealing histological features identified in **(B)**. **(D,E)** Immunostaining of Ki-67 and cleaved caspase-3, respectively, in the same ROI.

**Figure 3:**
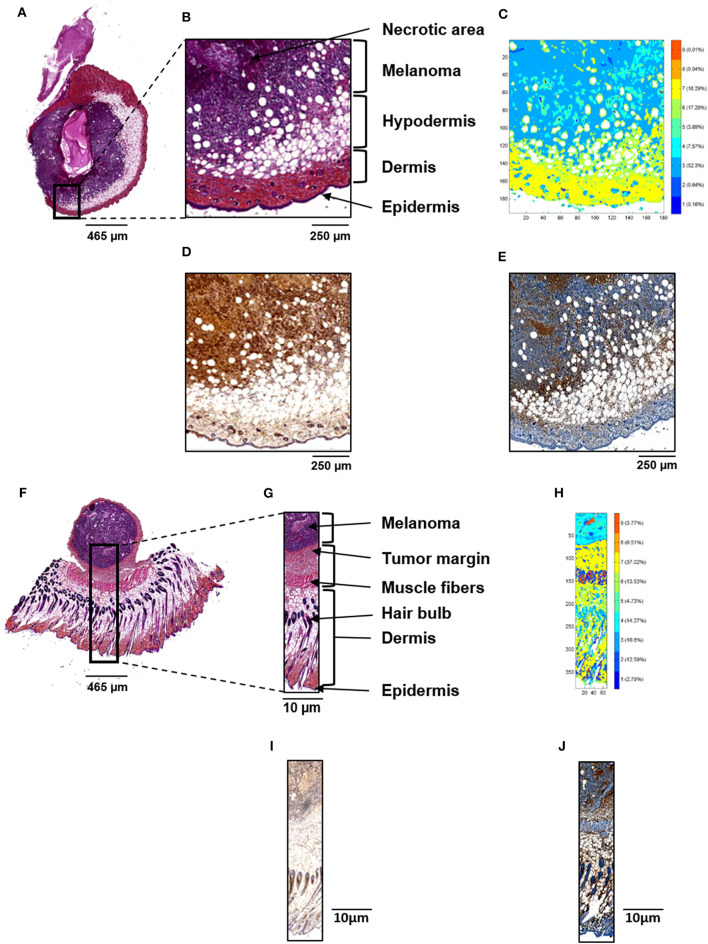
Conventional and spectral histologies of L9Mc SCR **(A–E)** and L9Mc-treated **(F–J)** melanoma primary tumor tissues. H&E staining **(A,F)** of the whole tissue sample. **(B,G)** Selected ROI (represented by a black rectangle in **A** and **F**, respectively) used for FTIR imaging. **(C,H)** Common K-means FTIR reconstructed image using 9 classes revealing histological features identified in **(B,G)**, respectively. Immunostaining of Ki-67 **(D,I)** and cleaved caspase-3 **(E,J)** in the same ROI.

Five control, five L9Mc SCR-treated and five L9Mc-treated melanoma primary tumor sections were examined by both conventional and infrared spectral histologies. H&E staining of representative sections of each group are shown in Figures 2A, 3A,F, respectively, and at a higher magnification (2.5x, 10x, and 15x) in Supplementary Figure 1 with the corresponding Crosscope links. In the case of L9Mc-treated melanoma, there is a clear evidence that the volume of the tumor is 3–4-fold reduced (*n* = 10, mean volume: 0.31 ± 0.26 cm^3^) compared to the control (*n* = 9, mean volume: 1.02 ± 0.97 cm^3^) and L9Mc SCR-treated (*n* = 8, mean volume: 1.25 ± 1.80 cm^3^) groups (data not shown). The regions of interest (ROI) analyzed by FTIR are represented by rectangles. In the case of control tissue, the ROI is highlighted in Figure 2B. The melanoma tumor is easily recognized by a purple staining, while peritumoral area appears in light pink staining. In addition, the muscle fibers are characterized by an intense pink color. Hair bulbs can be identified by their dark pink staining in the dermis. The highlighted zone of L9Mc SCR sections is shown in Figure 3B. Tumor cells are clearly visible in purple while necrotic area appears in light purple. Moreover, the external thin layer of the epidermis is characterized by a dark pink color, and the dermis appears in intense pink. The hypodermis layer is mainly characterized by a light pink staining. The highlighted zone of L9Mc sections is shown in Figure 3G. Similar histological structures can be assigned to this tissue. However, the ECM remodeling is less marked than in the case of control and L9Mc SCR-treated tumors.

**Figure 4:**
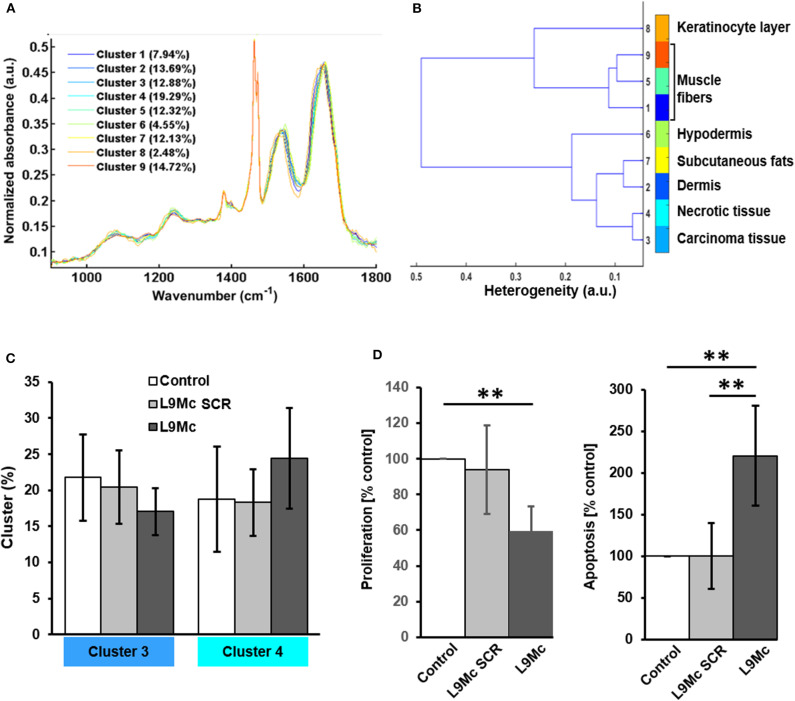
Centroid spectra **(A)** and dendrogram **(B)** resulting from common K-means analysis using 9 classes, each corresponding to different skin and carcinoma histological features. **(C)** Histogram showing the percentage contribution of clusters 3 (carcinoma tissue) and 4 (necrotic tissue) after L9Mc treatment, compared to L9Mc SCR treatment and control melanoma. Data represent the mean ± SE. **(D)** Histogram showing the percentage of positive cells for Ki-67 and cleaved caspase-3 by immunohistochemistry after L9Mc treatment, compared to L9Mc SCR treatment and control melanoma. Data represent the mean ± SD. A *P* < 0.05 was considered significant with **P < 0.01 as determined by the Student's *t*–test.

After digital dewaxing of the FTIR images of melanoma primary tumor tissue sections, a common K-means clustering was performed using 9 clusters. The common K-means is used here so that the same histological feature is assigned the same pseudo-color in all images. The reconstructed color-coded cluster images enabled the recovery of different histological features that allowed to precisely localize melanoma from other tissue components. These are depicted in Figures 2C, 3C,H for control, L9Mc SCR-treated and L9Mc-treated, respectively. The centroid spectra of the 9 clusters are displayed in Figure 4A. These centroid spectra allowed generating a hierarchical classification based on spectral distance (Figure 4B) and to visualize the similarities or differences between the different tissue components. Annotation of each generated cluster was then performed with the help of a confirmed pathologist resulting in the following precise tissue characterization: melanoma tissues were represented by cluster 3, necrotic tissues by cluster 4, epidermis by cluster 8, dermis with hair bulb by cluster 2, hypodermis and subcutaneous fats by clusters 6 and 7, respectively, and dermal muscle fibers by clusters 1, 5, and 9.

Further, we focused on clusters 3 and 4 (representing melanoma tissues and necrotic tissues, respectively) to evaluate the effect of the L9Mc peptide on tumor growth, as they seem to represent good qualitative spectral markers of melanoma tumors. The percentage of contribution of these 2 clusters in each spectral image was assessed for the 3 groups of mice and is shown in Figure 4C. Our data show that, in presence of L9Mc, melanoma tissue tends to decrease, while necrotic tissue tends to increase.

### L9Mc Decreases Proliferation and Increases Apoptosis of B16F1 Cells *in vivo*

The histological characterization of melanoma was further investigated by immunohistochemistry analysis for Ki-67 staining (control, *n* = 6; L9Mc SCR, *n* = 4; L9Mc, *n* = 4) and for cleaved caspase-3 staining (control, *n* = 6; L9Mc SCR, *n* = 5; L9Mc, *n* = 5). Ki-67 immunostaining for proliferation of B16F1 cells are shown in Figures 2D, 3D,I for control, L9Mc SCR and L9Mc, respectively, and at higher magnification (40x) in Supplementary Figure 2. Cleaved caspase-3 immunostaining for apoptosis of B16F1 cells are shown in Figures 2E, 3E,J for control, L9Mc SCR and L9Mc, respectively, and at higher magnification (40x) in Supplementary Figure 2.

Interestingly, the inhibitory effect of L9Mc on primary melanoma tumor volume is associated with a significant decrease in Ki-67 proliferation marker expression (59.44 ± 13.83% of positive cells) and increase in cleaved caspase-3 apoptosis marker (220.54 ± 59.87% of positive cells) while in the control and L9Mc SCR groups, the Ki-67 expression of positive cells were 100% and 93.75 ± 24.87% and the cleaved caspase-3 marker were 100% and 100.33 ± 39.58% of positive cells, respectively (Figure 4D). All data from statistical analyses are not shown here but we give below an example as an illustration.

Immunostaining results of melanoma primary tumor from control B16F1 cells showed high percentage of Ki-67 positive cells (82%) and low percentage of cleaved caspase-3 (3%) positive cells (Figures 2D,E, respectively). The K-means cluster image shown in Figure 2C correlates quite well with both histological and immunohistochemical data. Immunostaining results of melanoma primary tumor from L9Mc SCR and L9Mc showed 66 and 56% of Ki-67 positive cells (Figures 3D,I) and 1 and 40% of cleaved caspase-3 (Figures 3E,J), respectively. In a similar way as above, the K-means cluster images shown in Figures 3C,H correlate quite well with both histological and immunohistochemical data showing the advantage of label-free spectral histology.

## Discussion

Cancer diagnosis is mainly based on microscopic examination of stained tissue sections by an expert pathologist. This pathological examination depends on the cell morphology and tissue architecture. However, an accurate diagnosis of the cancer and its staging can be challenging (Kumar et al., 2018). There is an ongoing quest for an accurate, rapid, sensitive, and inexpensive method for cancer diagnosis. FTIR imaging can be a potential approach as it exhibits such characteristics and is moreover a label-free technique. It has proved to be useful to probe skin pathophysiological changes. For example, modifications of dermal collagen during chronological aging can be monitored by polarized FTIR imaging (Nguyen et al., 2014; Eklouh-Molinier et al., 2015). Pathological processes in skin can be characterized by FTIR imaging by identifying melanoma cells and tissues (Wald and Goormaghtigh, 2015; Wald et al., 2016b), discriminating between nevus and melanoma (Hammody et al., 2005; Tfayli et al., 2005), primary cutaneous melanoma (Ly et al., 2010), different metastatic forms (Andleeb et al., 2018) and different types of inflammatory skin lesions (Sebiskveradze et al., 2018). In combination with pattern recognition techniques, FTIR imaging was able to investigate tumor heterogeneity (Sebiskveradze et al., 2011) and differential diagnosis (Ly et al., 2009) in skin carcinoma. All these and other studies have clearly demonstrated the potential of IRSI as a non-invasive and non-destructive approach to investigate skin pathologies. The approach has also shown its potential for investigating other organs than skin. Using a robust prediction model, it has successfully differentiated normal and malignant colonic features without *a priori* histopathological information. The obtained IRSH images not only revealed common histology features, but also highlighted additional features like tumor budding and tumor- associated stroma (Nallala et al., 2014). Kuepper et al. studied UICC-Stage II and III colorectal cancers on 110 cases and reported very high sensitivity and specificity (Kuepper et al., 2018). In breast cancer it has shown its capability to delineate between non-inflammatory and inflammatory biopsies, the latter having a poor prognosis because of the lack of specific biomarkers (Mohamed et al., 2018). Using prostate tissue microarrays, Kwak et al. have reported area under ROC curve as high as 0.95 with a blind testing (Kwak et al., 2011). The potential of IRSH was taken a step further in a recent study where a score of tumor aggressiveness could be associated to preneoplastic lesions and squamous cell lung carcinomas. The score correlated quite well with the aggressiveness score calculated using histopathological criteria (Gaydou et al., 2018).

We have previously shown that melanoma progression was downregulated by lumican (Brézillon et al., 2013) and its derived peptides (Pietraszek et al., 2013). However, the role of lumican in cancer is controversial and in many cases it actually facilitates cancer growth. As an example of positive correlation, in lung adenocarcinomas the expression level of lumican in cancer cells correlated with pleural invasion and larger tumor size (Matsuda et al., 2008). Similarly, lumican overexpression in pancreatic cancer increased cell invasiveness and proliferation (Williams et al., 2010; Yamamoto et al., 2012; Sharma et al., 2013). In contrast, lumican was reported to decrease cell proliferation in osteosarcoma (Nikitovic et al., 2008). Lumican present in the ECM has restrictive role on invasion in prostate cancer (Coulson-Thomas et al., 2013), in melanoma (Stasiak et al., 2016), and in breast cancer (Troup et al., 2003; Karamanou et al., 2017, 2020).

In this report, our aim was to assess by FTIR imaging the effect of antitumoral effectors, such as the L9Mc lumican-derived peptide on primary melanoma development. In addition to its properties of melanoma tumor growth inhibition (Stasiak et al., 2016), lumican was previously described as a key actor in tumor matrix assembly *in vivo* (Jeanne et al., 2017). Thus, lumican was able to modulate the response of a therapeutic peptide targeting the extracellular matrix by specific inhibition of thrombospondin-1, playing a substantial role in maintaining tumor microenvironment integrity (Jeanne et al., 2017). In addition, lumican has been shown to inhibit lung metastasis by decreasing cell proliferation and by stimulating cell apoptosis of melanoma nodules (Brézillon et al., 2009). Moreover, the effect of lumican-derived peptides on growth and migration of melanoma cells was previously demonstrated *in vitro*. Lumcorin and L9M peptides, which contain the same LRR9 core sequence as L9Mc, have been described to inhibit melanoma cell migration in a mechanism including focal adhesion kinase (FAK) dephosphorylation and matrix metalloproteinase-14 (MMP-14) inhibition. Furthermore, these two peptides were shown to significantly decrease proliferation of melanoma cells and their ability to form colonies in soft agar assay (Pietraszek et al., 2013).

After initiating melanoma primary tumors via injecting control, L9Mc SCR and L9Mc peptides treated B16F1 melanoma cells, the obtained melanoma tissues underwent FTIR imaging analysis. Using common K-means cluster analysis with 9 clusters, melanoma, and normal skin tissue structures were clearly distinguished *via* the obtained color-encoded images. This is also evidenced by the dendrogram obtained after hierarchical cluster analysis of the centroid spectra, based on similarity evaluation using Ward's algorithm. This correlates well with the results obtained by H&E staining, highlighting the potential of spectral histology in tissue characterization.

Comparison of cluster percentages between control, L9Mc SCR- and L9Mc-treated melanoma tumors showed no significant statistical differences. However, a decrease trend in the contribution of cluster 3 (melanoma tissues) was observed in presence of L9Mc compared to control and L9Mc SCR. In a similar manner, we observed an increase trend for cluster 4 (necrotic tissues) in L9Mc-treated melanoma tissues compared to control and L9Mc SCR-treated melanoma tissues.

In order to understand and complement these observed spectral trends, IHC analysis was performed using Ki-67 proliferation and cleaved caspase-3 apoptotic markers. The percentage of positive cells for Ki-67 staining was significantly decreased in L9Mc-treated melanoma tumors compared to the control melanoma tissues, showing the anti-proliferative activity of this peptide. Interestingly, the L9M peptide was shown to inhibit melanoma cell proliferation (Pietraszek et al., 2013). This is in accordance with the melanoma *in vivo* model used in this present study, highlighting L9Mc as having a similar biological effect as lumcorin and L9M. On the other hand, the percentage of positive cells for cleaved caspase-3 showed a significant increase in the L9Mc-treated melanoma tumors compared to the control and L9Mc SCR-treated melanoma tissues, highlighting the pro-apoptotic effect of this peptide. Interestingly, lumican was previously shown to reduce melanoma tumor growth through the induction of apoptosis (Vuillermoz et al., 2004). Proliferation and apoptosis mechanisms of action of lumican were elucidated by the characterization of lumican-deficient mice. In lumican knockout Lum^−/−^ mice, apoptosis of stromal cells was down-regulated. The function of FasL on intra-ocular tumors was determined by the microenvironment in conjunction with the form and level of FasL expressed (Gregory et al., 2002). The Lum^−/−^ fibroblasts exhibited a decreased p21WAF/CIP1 expression, an universal inhibitor of cyclin-dependent kinases, and consequently increased cyclins A, D1, and E. The tumor suppressor p53, an upstream regulator of p21, is down-regulated in Lum^−/−^ fibroblasts. Thus, the regulation of p21 by lumican is a p53-dependent pathway (Vij et al., 2004). Lumican overexpression was shown to suppress tumorigenic transformation of rat fibroblasts induced by v-src and v-K-*ras* (Yoshioka et al., 2000). Lumican overexpression decreases subcutaneous primary melanoma tumor growth *in vivo*, with a concomitant decrease of cyclin D1 expression (Vuillermoz et al., 2004) as well as a decrease in the number of lung metastatic nodules in which an increase of tumor cell apoptosis was observed.

Lumican and its derived peptides were also demonstrated to inhibit melanoma cell proliferation *in vitro* (D'Onofrio et al., 2008; Zeltz et al., 2009; Brézillon et al., 2013; Pietraszek et al., 2013; Stasiak et al., 2016; Jeanne et al., 2017).

Based on these immunohistochemistry results, clusters 3 and 4 could be identified as potential spectral markers to study the effect of anti-tumor peptides on proliferation and apoptosis.

In the context of this study, the IRSH images were performed at 6.25 μm/pixel available with our instrumentation. It may be argued that this can be a limitation to correctly perform digital histopathology. This is indeed not a limitation to the technique because it has been previously shown that optics upgrade allows imaging with an effective geometric pixel size of ~1 × 1 μm^2^ (Findlay et al., 2015) and high contrast stain-free digital histopathology has been reported with smaller pixel size of 0.32 × 0.32 μm^2^ (Schnell et al., 2020). One shortcoming impeding the clinical translation of conventional IRSH has been the fact that it is too time-consuming for consideration as a clinical tool. This can now be circumvented due to recent instrumental development based on infrared quantum cascade lasers (QCL). In this context, IRSH is able to provide highly resolved diagnostic images with short acquisition times, in the time-frame equivalent to frozen sample handling by the pathologist (Yeh et al., 2015; Kuepper et al., 2018). On a clinical ground, it does not mean that the technique will replace conventional histology but an all-digital histopathology could be of an aid to pathologists for rapid screening of biopsies. Thus, a trained system with a good database and machine learning approaches is able to correctly and objectively identify histological features and perform digital IRSH with high sensitivity and specificity (AUC ≥0.95). This has been demonstrated in several studies (Kwak et al., 2011; Kuepper et al., 2018).

## Conclusion

Melanoma is one of the most lethal and fatal forms of skin cancer with a higher incidence of metastasis. Understanding these pathological conditions is crucial for patient therapy and management. In this study, we show that FTIR imaging is a potential tool to investigate changes occurring in melanoma tissues treated with peptide-based anti-tumor molecules. Therefore, such a novel approach based on spectral analysis can complement conventional histology and immunohistochemistry techniques with the advantage of being rapid, non-destructive, reagent-, and label-free.

## Data Availability Statement

All datasets generated for this study are included in the article/Supplementary Material.

## Ethics Statement

All experiments were performed according to the instructions of the Center National de la Recherche Scientifique. This study was performed in compliance with the French Animal Welfare Act and following the French Board for Animal Experiments. Experiments were conducted under approval of the French Ministère de l'Enseignement Supérieur et de la Recherche (Ethics Committee C2EA-56) in accordance with the directive 2010/63/UE.

## Author Contributions

SB and GS contributed to study conception and design. CB-R, SB, VU, PN, HM, and GS contributed to manuscript writing and revision. EA, HM, VU, IP, and SB performed experiments. CB-R, SB, VU, HM, and GS contributed to data analysis and interpretation.

## Conflict of Interest

The authors declare that the research was conducted in the absence of any commercial or financial relationships that could be construed as a potential conflict of interest.

## Supplementary Material

Supplementary Figure 1 Highlight on skin histological structures. H&E stained sections (2.5x, 10x, and 15x) of B16F1 primary melanoma tumors are shown for control, L9Mc SCR and L9Mc treated mice, respectively. Hematoxylin and Eosin solution highlights the nucleus in purple and the cytoplasm in pink, respectively. The stained sections are available via the following Crosscope links: - Control https://accounts.crosscope.com/quorum/4d05469771f2506a7b3d5277f87e97a3 - L9M SRC https://accounts.crosscope.com/quorum/eb1abb0ea6a108bc3844eeb00b091070 - L9Mc https://accounts.crosscope.com/quorum/af01e8c842752f8b2572bfed6ea7eb3f

Supplementary Figure 2 Cleaved caspase-3 and Ki-67 immunostaining. Representative immunostaining (40x) of cleaved caspase-3 (top row) and Ki-67 (bottom row) of B16F1 cells are shown for control (left column), L9Mc SCR (middle column) and L9Mc treated mice (right column), respectively. Cleaved caspase-3, marker of apoptosis, and Ki-67, marker of proliferation, are detected in brown in the nucleus and in the cytoplasm, respectively. As compared to both control groups, an increased apoptosis and a decreased proliferation are observed in L9Mc treated mice.
