# Supplementary figures and images for: Label-Free Infrared Spectral Histology of Skin Tissue Part II: Impact of a Lumican-Derived Peptide on Melanoma Growth
Source: Front Cell Dev Biol. 2020 May 29;8:377. doi: 10.3389/fcell.2020.00377 (PMC7273845; doi:10.3389/fcell.2020.00377)

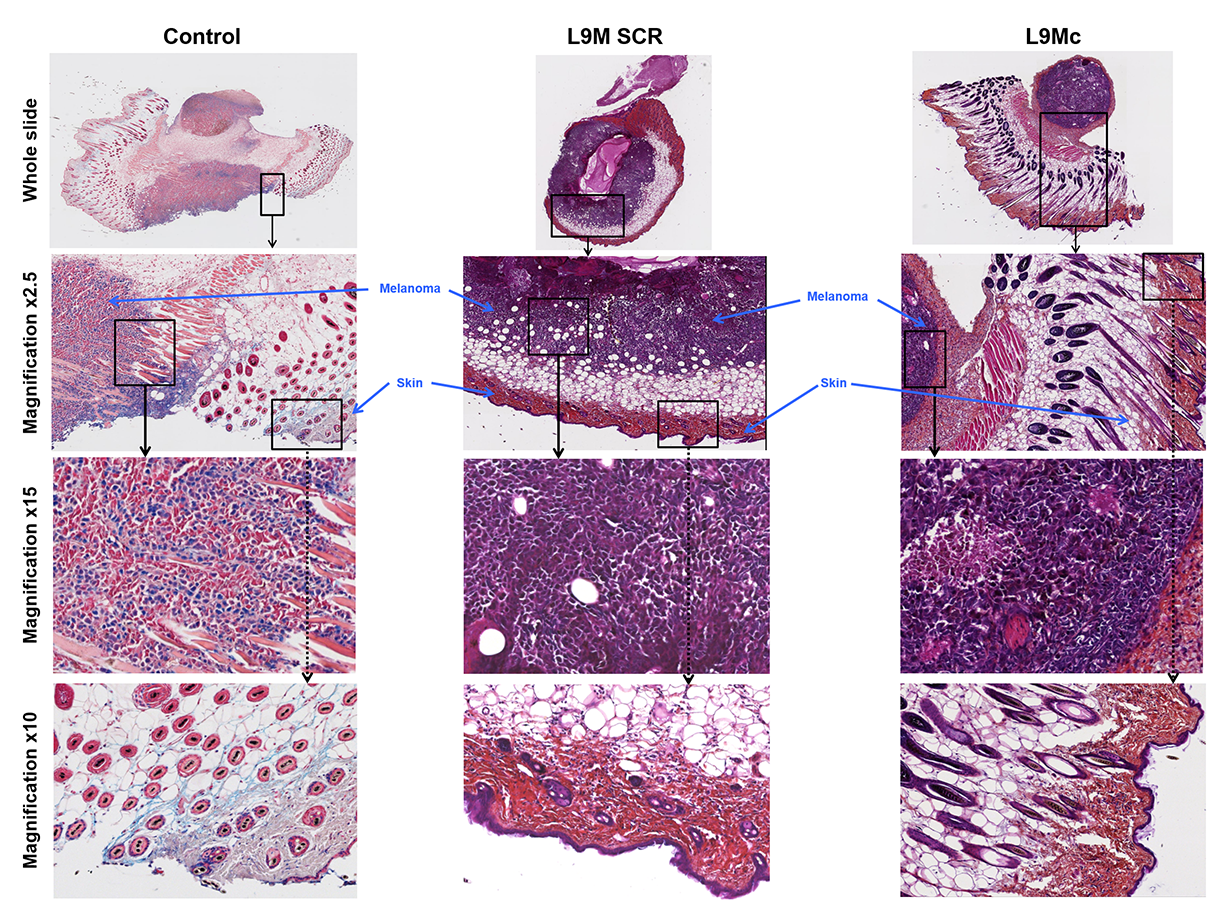

Supplement: Supplementary Figure 1 — Highlight on skin histological structures. H&E stained sections (2.5x, 10x, and 15x) of B16F1 primary melanoma tumors are shown for control, L9Mc SCR and L9Mc treated mice, respectively. Hematoxylin and Eosin solution highlights the nucleus in purple and the cytoplasm in pink, respectively. The stained sections are available via the following Crosscope links: - Control https://accounts.crosscope.com/quorum/4d05469771f2506a7b3d5277f87e97a3 - L9M SRC https://accounts.crosscope.com/quorum/eb1abb0ea6a108bc3844eeb00b091070 - L9Mc https://accounts.crosscope.com/quorum/af01e8c842752f8b2572bfed6ea7eb3f [file Image_1.TIF]

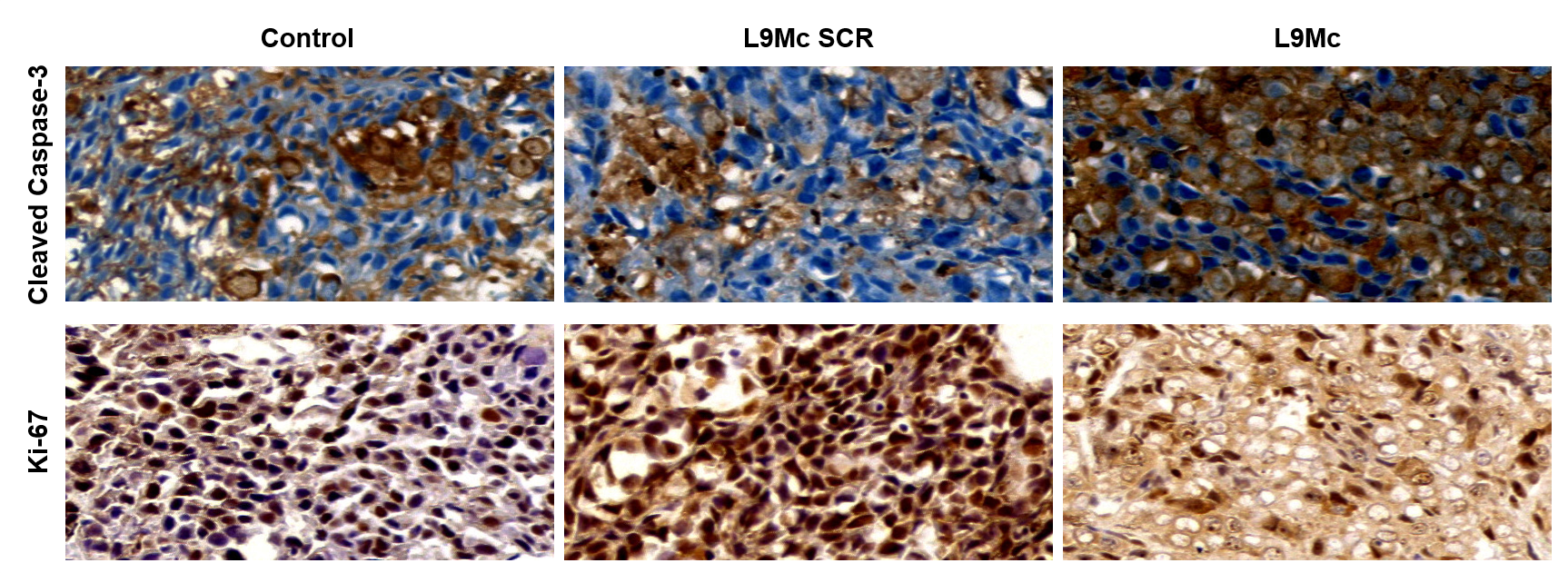

Supplement: Supplementary Figure 2 — Cleaved caspase-3 and Ki-67 immunostaining. Representative immunostaining (40x) of cleaved caspase-3 (top row) and Ki-67 (bottom row) of B16F1 cells are shown for control (left column), L9Mc SCR (middle column) and L9Mc treated mice (right column), respectively. Cleaved caspase-3, marker of apoptosis, and Ki-67, marker of proliferation, are detected in brown in the nucleus and in the cytoplasm, respectively. As compared to both control groups, an increased apoptosis and a decreased proliferation are observed in L9Mc treated mice. [file Image_2.tif]
